# Pyroptosis: An Accomplice in the Induction of Multisystem Complications Triggered by Obstructive Sleep Apnea

**DOI:** 10.3390/biom14111349

**Published:** 2024-10-23

**Authors:** Jingwen Han, Lisong Ye, Yan Wang

**Affiliations:** 1 Department of Orthodontics, Shanghai Stomatological Hospital & School of Stomatology, Fudan University, Shanghai 200001, China; jwhan24@m.fudan.edu.cn (J.H.); lsye23@m.fudan.edu.cn (L.Y.); 2 Shanghai Key Laboratory of Craniomaxillofacial Development and Diseases, Fudan University, Shanghai 200001, China; 3 Department of Preventive Dentistry, Shanghai Stomatological Hospital & School of Stomatology, Fudan University, Shanghai 200001, China

**Keywords:** obstructive sleep apnea, pyroptosis, chronic intermittent hypoxia, sleep deprivation, NLRP3 inflammasome

## Abstract

Obstructive sleep apnea (OSA) is a common respiratory disorder, primarily characterized by two pathological features: chronic intermittent hypoxia (CIH) and sleep deprivation (SD). OSA has been identified as a risk factor for numerous diseases, and the inflammatory response related to programmed cell necrosis is believed to play a significant role in the occurrence and progression of multisystem damage induced by OSA, with increasing attention being paid to pyroptosis. Recent studies have indicated that OSA can elevate oxidative stress levels in the body, activating the process of pyroptosis within different tissues, ultimately accelerating organ dysfunction. However, the molecular mechanisms of pyroptosis in the multisystem damage induced by OSA remain unclear. Therefore, this review focuses on four major systems that have received concentrated attention in existing research in order to explore the role of pyroptosis in promoting renal diseases, cardiovascular diseases, neurocognitive diseases, and skin diseases in OSA patients. Furthermore, we provide a comprehensive overview of methods for inhibiting pyroptosis at different molecular levels, with the goal of identifying viable targets and therapeutic strategies for addressing OSA-related complications.

## 1. Introduction

Obstructive sleep apnea (OSA), the most common type of sleep-disordered breathing (SDB) [1], and involves recurrent airway collapse lasting 10 s or more during sleep, causing either hypopnea (reduced airflow) or apnea (absence of airflow) [2]. The two main manifestations of OSA are chronic intermittent hypoxia (CIH) and sleep deprivation (SD). Repeated episodes of intermittent hypoxia and reoxygenation can impact the mitochondrial electron transport chain, resulting in the excessive production of reactive oxygen species (ROS) [3], while prolonged sleep deprivation can disrupt the balance of antioxidant oxidation products, influence the synthesis and metabolism of various cytokines, and aggravate the hypoxia process [4]. OSA has emerged as a global health burden, not only disrupting nighttime respiration and fragmenting sleep, but also increasing the risk of traffic and occupational accidents [5]. Moreover, OSA can trigger multisystem damage. It serves as an independent risk factor for several diseases, including urinary system diseases (such as chronic kidney disease and acute kidney injury) [4,6,7], cardiovascular diseases (such as atherosclerosis, hypertension, and arrhythmias) [8,9], central nervous system diseases (such as Parkinson’s disease and Alzheimer’s disease) [10,11,12], and metabolic diseases (such as diabetes and gout) [13,14]. Although the exact mechanisms are not fully understood, numerous studies indicate that inflammation is a key factor in the development of OSA-related complications.

Pyroptosis is a kind of inflammatory programmed cell death executed by the protein family of gasdermins. Activated by the cysteinyl aspartate specific proteinase (caspase), gasdermin family proteins punch pores in plasma membranes, leading to capsule rupture and the release of interleukin (IL)-1β and IL-18, triggering inflammatory cascades [15]. Pyroptosis constitutes a crucial innate immune response in the human body; under normal circumstances, pyroptosis triggers the immune system to eliminate pathogens present within the host [16]. However, excessive activation of pyroptosis can induce inflammatory cytokine storm, resulting in pathological changes to various diseases. With the loss of cellular integrity, more damage-associated molecular patterns (DAMPs) are released from pyroptotic cells, including IL-1α, high mobility group box 1 (HMGB1), and ATP, which further activate the immune response [17]. Extensive evidence indicates that aberrant pyroptosis contributes to the pathogenesis of various diseases including, but not limited to, infectious diseases, autoimmune diseases, and tumors [18]. Elevated oxidative stress levels in individuals with OSA can activate the pyroptotic process in different tissues, leading to impaired organismal function [19,20,21]. This suggests that pyroptosis may serve as an important intrinsic mechanism linking OSA to its complications.

In this review, we discuss the involvement of pyroptosis in the progression of complications in OSA patients. Given that the current research on pyroptosis in OSA comorbidities mainly focuses on renal diseases, cardiovascular diseases, neurocognitive disorders, and skin diseases, this article primarily discusses these four aspects. Furthermore, we explore potential strategies to inhibit pyroptosis in OSA patients. We firmly believe that directly treating OSA is undoubtedly the most fundamental approach to preventing its multi-organ complications, and the current clinical treatments for OSA (such as improving behavioral patterns, using oral appliances, and undergoing upper airway surgery [2]) are likely to indirectly reduce OSA-related pyroptosis through the inhibition of CIH and/or SD. This review aims to identify the pyroptotic mechanisms linking OSA with its systemic complications and to explore drugs or other therapeutic approaches targeting molecular pathways directly associated with these mechanisms, as adjuncts to clinical OSA treatment, to reduce the systemic damage caused by OSA. As such, we do not delve into the details of clinical OSA treatments unless they have been approved to directly inhibit OSA-related pyroptosis pathways.

## 2. Pyroptosis: A Type of Programmed Cell Necrosis

Since the discovery of interleukin-1 beta converting enzyme (ICE, later renamed as Caspase1) [22,23], a form of cell death dependent on Caspase1 and accompanied by an inflammatory response has long been defined as apoptosis [24,25]. Until 2000, Brennan et al. observed distinct differences in macrophage death following Salmonella infection compared to conventional apoptosis [26]. This newly identified form of cell death, defined by membrane rupture, intense inflammation, and reliance on Caspases, was eventually named “pyroptosis” [27].

In the canonical pathway, pattern recognition receptors (PRRs) identify pathogen-associated molecular patterns (PAMPs) and DAMPs to activate inflammasomes. Inflammasomes are cytoplasmic multiprotein complexes composed of PRRs, apoptosis-associated speck-like protein (ASC), and pro-Caspase1. Currently known PRRs are primarily NOD-like receptor (NLR) family proteins and pyrin and HIN domain-containing protein (PYHIN) family proteins, including NLRP1, CARD8, NLRP3, NLRC4/NAIP, AIM2, Pyrin, and NLRP6. ASC functions as an adaptor protein, comprising a pyrin domain (PYD) and a caspase activation and recruitment domain (CARD). However, ASC is not obligatory for the recruitment of pro-Caspase. PYD-containing proteins such as NLRP3, AIM2, NLRP6, and Pyrin recruit ASC, which then recruits pro-Caspase1 through CARD–CARD interactions, while NLRP1 and CARD8 possess a CARD domain, allowing their direct binding to pro-Caspase1 via CARD–CARD interaction [28]. Research indicates that in patients with severe OSA, the activation of the NLRP3 inflammasome and the canonical pathway of pyroptosis that it mediates may serve as a significant linking mechanism between OSA pathology and systemic inflammatory response [29]. It has now been established that the NLRP3 inflammasome requires a two-checkpoint activation mechanism, including a priming step and an assembly step. The priming signal activates Toll-like receptors (TLRs) or cytokine receptors, initiating the NF-κB signaling pathway to upregulate the transcription and translation of NLRP3 and cytokine precursors. This process may also induce post-translational modifications of NLRP3. The assembly step is triggered by various stimuli and cellular events, such as ROS, extracellular ATP, bacterial toxins, insoluble particles, and pathogenic microorganisms, leading to the promotion of inflammasome assembly [30,31]. After inflammasome activation, pro-Caspase1 undergoes hydrolysis and oligomerization to form dimers (p20/p10) and become active Caspase1. Activated Caspase1 can cleave pro-IL-1β and pro-IL-18, converting them into mature IL-1β and IL-18, respectively. On the other hand, Caspase1 cleaves gasdermin D (GSDMD) at Asp275 (Asp276 in mice), releasing its amino-terminal N-terminal domain (GSDMD-NT) which is inhibited by the carboxy-terminal C-terminal domain (GSDMD-CT) [32]. GSDMD-NT undergoes palmitoylation at Cys191/Cys192 (human/mouse) and then binds to phosphatidylinositol phosphates and phosphatidylserine restricted to the inner leaflet of the plasma membrane, forming non-selective transmembrane pores of about 12–14 nm in inner diameter. These pores lead to cell death and trigger the release of inflammatory mediators [33,34,35,36,37].

Unlike the canonical pathway, human pro-Caspase4/5 and mouse pro-Caspase11 can directly bind to the lipid A moiety in cytoplasmic lipopolysaccharide (LPS) through the CARD domain to form “noncanonical inflammasomes” and activate, without relying on other intracellular receptors [38]. In this case, Caspase4/5/11 simultaneously act as PRRs and pyroptosis-inducing effector proteins [28]. The cleavage mechanism of GSDMD by Caspase4/5/11 is similar to that of Caspase1. However, Caspase4/5/11 cannot directly process pro-IL-1β and pro-IL-18, but Caspase11 activation can lead to a low level of IL-1β secretion in a manner dependent on NLRP3 inflammasome [39].

In addition to GSDMD, the gasdermin family comprises other members, including GSDMA, GSDMB, GSDMC, GSDME (also known as DFNA5), and DFNB59, with a sequence homology of approximately 45%. Except for DFNB59, which lacks the C-terminal domain, all gasdermin proteins adopt a GSDMD-like two-domain architecture and are capable of inducing pyroptosis in a similar manner [39]. GSDMA is highly expressed in the skin and upper gastrointestinal epithelium, primarily inducing keratinocyte pyroptosis and playing a crucial role in defending against group A Streptococcus (GAS) infections [40]. GSDMB is expressed in humans but not in mice, with five reported subtypes: GSDMB3 and GSDMB4 can induce pyroptosis in tumor cells, while GSDMB1, GSDMB2, and GSDMB5 inhibit the cytotoxic effects of GSDMB3 and GSDMB4 [41]. GSDMC is highly expressed in the gastrointestinal tract and also participates in anti-tumor immunity; however, its role as a tumor suppressor or promoter remains uncertain, potentially related to the duration and intensity of the inflammatory response [42]. GSDMD and GSDME exhibit a broader expression profile, with high levels found in immune cells like macrophages and dendritic cells, highlighting their important roles in host defense and immunity [43].

**Figure 1:**
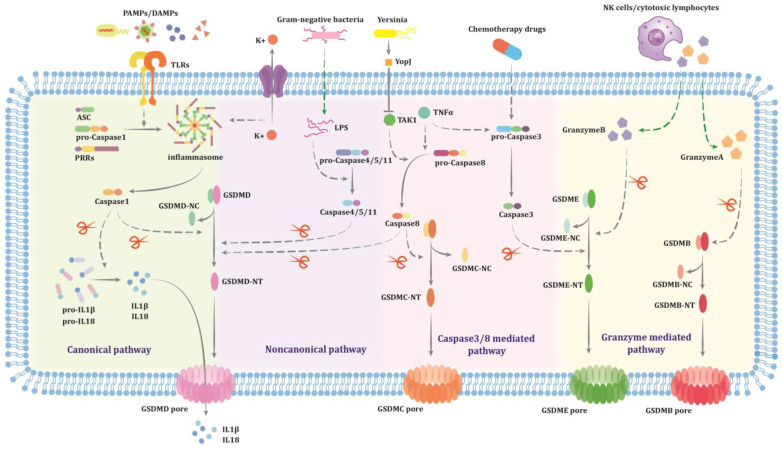
Mechanisms of pyroptosis.

Caspase4/5/11, mentioned above, are referred to as inflammatory caspases. Subsequent studies have revealed that Caspase8 and Caspase3, which mediate apoptosis, can also participate in the process of pyroptosis by cleaving GSDMC/GSDMD and GSDME, respectively [44,45,46,47,48]. Caspase8 can also indirectly act on GSDME by activating Caspase3 and Caspase7 [47]. The occurrence of apoptosis or pyroptosis in cells is determined by the substrates hydrolyzed by Caspases, specifically the abundance of gasdermin proteins. Moreover, the cleavage of gasdermin proteins is not exclusively mediated by Caspases. For example, streptococcal pyrogenic exotoxin B (SpeB) can cleave GSDMA, inducing keratinocyte pyroptosis [40]. Granzyme also mediates pyroptosis, with GSDMB and GSDME being cleaved by Granzyme A and Granzyme B, respectively [49,50]. The Nomenclature Committee on Cell Death (NCCD) defines pyroptosis as a type of regulated cell death (RCD) that primarily relies on the formation of plasma membrane pores by the gasdermin protein family, frequently, though not exclusively, due to inflammatory caspase activation [51]. The processes of different types of pyroptosis are illustrated in Figure 1.

The latest research indicates that mitochondrial damage caused by GSDMD-NT is likely a “point of no return” for pyroptosis. Before membrane rupture, GSDMD-NT binds to mitochondrial cardiolipin, disrupting both inner and outer membranes. This leads to a reduction in mitochondrial numbers, increased mitophagy, elevated ROS production, loss of transmembrane potential, decreased oxidative phosphorylation (OXPHOS), and the release of mitochondrial proteins and DNA from both the matrix and the intermembrane space. Other gasdermin proteins can also trigger this mitochondrial damage pathway. In certain instances, cells exhibit a phenomenon called “hyperactivation”, wherein they survive inflammasome activation and GSDMD-NT pore formation while still releasing inflammatory mediators. This may be associated with limited mitochondrial damage [52]. Therefore, the state of mitochondrial damage deserves more attention in future studies on pyroptosis. A comprehensive understanding of cellular responses to mitochondrial damage under various conditions will contribute to a thorough comprehension of pyroptosis. This will provide further insights and opportunities for developing future therapeutic strategies for related diseases.

## 3. Pyroptosis Promotes Multisystem Complications in OSA Patients

### 3.1. The Promotive Role of Pyroptosis in Renal Diseases of OSA Patients

OSA is thought to be linked to the development and progression of chronic kidney disease (CKD) [6,7,53] and acute kidney injury (AKI) [54,55], with pyroptosis playing a crucial role therein. The tubular injury induced by renal tubular epithelial cells (RTECs) in pyroptosis is usually an early event during AKI. Subsequently, the pyroptotic RTECs release pro-inflammatory factors and DAMPs that enhance the inflammatory response, driving a second wave of RTEC death and leading to sustained decline in renal function [56,57,58].The irreversible loss of RTECs during the acute phase can prompt remaining tubular cells to adapt to increased metabolic demands through stress response pathways, inducing tubulointerstitial inflammation and renal fibrosis, further progressing to CKD [59].

In vivo experiments have shown that CIH, a key pathological feature of OSA, can cause significant pathological changes in rat renal tissues, marked by extensive infiltration of inflammatory cells. Following CIH treatment, the expression levels of IL-1β, IL-18, lactate dehydrogenase (LDH), and ROS were observed to be markedly elevated, and NLRP3, Caspase1, GSDMD, TLR4, MyD88, p-NF-κB, and NF-κB were also significantly upregulated, positively correlating with the degree of hypoxia. These findings suggest that CIH activates the NLRP3 inflammasome pathway in renal tissues, inducing the production of inflammatory factors and triggering RTEC pyroptosis [20]. In CIH-induced RTEC injury, activation of TLR4 induces phosphorylation and degradation of I-κB through MyD88, further triggering NF-κB activation and its translocation from the cytoplasm to the nucleus, thereby initiating transcription and expression of NLRP3 and pro-IL-1β [60], and the hypoxia-induced ROS are likely to serve as the second signal for activation of the NLRP3 inflammasome [61]. In support of this, TLR4 deficiency has been demonstrated to attenuate the increased expression of MyD88 and NF-κB p65 after CIH treatment, and to alleviate renal histological damage and renal dysfunction [62]. Endoplasmic reticulum stress (ER) is also associated with CIH-induced RTEC pyroptosis. Exposure to CIH significantly increases the expression of ER stress markers glucose-regulated protein 78 (GRP78) and C/EBP homologous protein (CHOP), suggesting elevated ER stress levels [63]. Overactive ER activates the CHOP/Caspase11 signaling pathway, subsequently inducing RTEC pyroptosis in a Caspase1 dependent or independent manner [64]. MiRNAs play a crucial regulatory role in CIH-induced RTEC pyroptosis. CIH exposure induces *miR-155* expression both in vivo and in vitro [65], and *miR-155* directly represses FoxO3a expression and its downstream protein apoptosis repressor with caspase recruitment domain (ARC), thereby promoting RTEC pyroptosis [66]. Furthermore, CIH has been demonstrated to induce renal fibrosis [62], possibly associated with the release of high-mobility group box 1 (HMGB1) following RTEC pyroptosis, which triggers the migration and recruitment of monocytes/macrophages [67].

It has been reported that continuous hypoxia/reoxygenation stimuli could induce RTEC pyroptosis through the Caspase1/GSDMD axis and Caspase3/GSDME axis (with involvement of Caspase8 and Caspase9), along with inflammation in surrounding tissues [68,69]. While some scholars have likened CIH in OSA patients to the ischemia/reperfusion injury following acute myocardial infarction, it is noteworthy that ischemia/reperfusion and the simulated hypoxia/reoxygenation process described above occur relatively slowly over several hours to days, often as single events. In contrast, CIH induced by OSA typically lasts for seconds and occurs repetitively. Therefore, further comparative studies are needed to confirm whether cellular responses during ischemia/reperfusion and continuous hypoxia/reoxygenation processes are consistent with those observed in the context of CIH associated with OSA.

Based on the above findings, RTEC pyroptosis induced by OSA-related CIH is suggested as a mechanism of renal inflammatory injury, which may contribute to renal fibrosis. Future studies could investigate the mechanisms and regulatory networks underlying RTEC pyroptosis in OSA, aiming to identify precise intervention targets to improve the prevention and treatment of OSA-related renal damage. Additionally, pyroptosis has been implicated in other renal diseases, including diabetic kidney injury, renal cell carcinoma, and hyperuricemic nephropathy [70]. However, whether OSA accelerates the progression of these diseases through promoting pyroptosis remains unclear. Further exploration of OSA-related pyroptosis in various renal diseases may offer additional theoretical support for treating renal conditions in OSA patients.

### 3.2. The Promotive Role of Pyroptosis in Cardiovascular Diseases of OSA Patients

OSA is an independent risk factor for cardiovascular diseases [71]. As the pathological basis of cardiovascular and cerebrovascular diseases, atherosclerosis (AS) is a common comorbidity of OSA patients, mainly caused by the interaction of dyslipidemia and systemic inflammation [21]. As research on AS advances, it is increasingly viewed as a chronic inflammatory disease, with macrophage pyroptosis playing a more prominent role than traditional forms of programmed cell death (e.g., apoptosis and autophagy) in the formation, rupture, and immune–inflammatory responses of atherosclerotic plaques [72].

Oxidized low density lipoproteins (oxLDLs) are a class of active oxidant generated by the lipid peroxidation of LDL particles. They bind to macrophage CD36 through their lipid moiety, or to other scavenger receptors via their apoprotein moiety after being endocytosed by cells through a raft-mediated pathway. This process leads to foam cell formation and the development of AS pathogenesis [73,74,75]. In OSA patients, the generation of oxLDL may be associated with ROS production and elevated oxidative stress [76,77]. Research has confirmed that, even in the absence of other major risk factors, individuals with OSA exhibit higher plasma LDL levels. This elevation correlates with AHI (apnea-hypopnea index), time with oxygen saturation <90% (CT90), and oxygen desaturation index (ODI), and these associations are particularly evident in patients in the early stages of AS (prior to the appearance of atherosclerotic plaques), suggesting that oxidative stress induced by OSA and the resultant increase in oxLDL may play a critical role in the development of AS [21]. In vitro experiments have shown that CIH acts as a priming trigger for NLRP3 inflammasome activation in macrophages. This upregulates HIF-1α and activates the NF-κB signaling pathway, leading to increased expression levels of NLRP3, ASC, pro-Caspase1, GSDMD-NT, tissue factor (TF), and pro-inflammatory cytokine genes. Additionally, plasma oxLDL serves as an assembly signal for NLRP3 inflammasome via recognition by CD36 [21]. Furthermore, under normoxia conditions, oxLDL is sufficient to activate the NLRP3 inflammasome [21], and activate HIF-1α in monocytes in a hypoxia-independent manner [78], suggesting that plasma oxLDL can also serve as a priming trigger for NLRP3 inflammasome activation. This process likely depends on oxLDL binding to macrophage CD36, inducing the assembly of TLR4/TLR6 heterodimers and activating the NF-κB pathway [79]. Moreover, oxLDL uptake by CD36 leads to the formation of intracellular cholesterol crystals (CCs), causing lysosomal damage and triggering assembly and activation of the NLRP3 inflammasome. CCs also activate the complement system, promoting monocyte activation and CC phagocytosis, thereby activating NLRP3 inflammasome [80]. The above studies indicate that oxLDL and CIH synergistically contribute to NLRP3 inflammasome activation in OSA patients, ultimately leading to pyroptosis and the release of inflammatory cytokines and TF [21]. The proinflammatory cytokine IL-1β, released from macrophage pyroptosis induced by OSA, has been demonstrated to play multiple roles in the formation of atherothrombotic plaque, including inducing procoagulant activity, promoting the adhesion of monocytes and leukocytes to vascular endothelial cells, and stimulating the growth of vascular smooth-muscle cells [81], while IL-18 exacerbates AS by stimulating the development of Th1-type CD4+ T cells that secrete IFN-γ [82]. Additionally, IL-1β and IL-18 can initiate inflammasome activation through the IL-1 receptor (IL-1R) [80]. This creates a vicious cycle of inflammasome activation in AS. The actions of these inflammatory factors promote the progression of AS in OSA patients.

In addition to AS, OSA elevates the risk of other cardiovascular diseases by inducing pyroptosis. Macrophage pyroptosis, as mentioned earlier, contributes to the formation of lipid-rich pools and the necrotic core of plaques in advanced lesion stages, thereby increasing plaque instability and rupture risk [83]. After plaque rupture, exposed cellular pyroptosis fragments and cellular contents (such as TF) can serve as potent procoagulant and inflammatory stimuli, triggering thrombus formation, and potentially leading to myocardial infarction, ischemic heart disease, ischemic stroke, and other cerebrovascular diseases. Additionally, hypoxia-induced generation of ROS can lead to vascular endothelial cell pyroptosis, upregulation of adhesion molecule expression, and cytokine secretion, promoting recruitment of monocytes to the intima [83,84]. Similarly, ROS in myocardial ischemia-reperfusion also activates the NLRP3 inflammasome, inducing cardiomyocyte pyroptosis and exacerbating myocardial injury [85].

In summary, pyroptosis plays a significant promoting role in cardiovascular disease among OSA patients, potentially increasing cardiovascular event risk by promoting AS development and plaque instability. This provides new therapeutic targets for OSA-related cardiovascular diseases. Future research should further explore the specific mechanisms of pyroptosis in OSA-related cardiovascular diseases, particularly whether OSA can activate pyroptosis in endothelial cells, cardiomyocytes, fibroblasts, and other cells similar to ischemia-reperfusion. This investigation is crucial for identifying effective strategies to control OSA-related cardiovascular complications.

### 3.3. The Promotive Role of Pyroptosis in Neurocognitive Diseases of OSA Patients

Epidemiological studies have suggested that OSA is a significant risk factor for dementia [1], linked to an earlier onset of mild cognitive impairment (MCI) and Alzheimer’s disease (AD), as well as accelerated cognitive decline [86]. Currently, several explanations have been proposed regarding how OSA affects cognition, with increasing emphasis on the pathogenic role of neuroinflammation [15]. However, molecular events and regulatory mechanisms remain unclear. Recent studies indicate that CIH and SD can directly induce hippocampal neuronal pyroptosis and may lead to “hyperactivation” of microglia and increased levels of astrocyte pyroptosis. These processes can trigger tissue inflammation, exacerbate brain damage, and ultimately result in impaired cognitive function [19,87,88,89].

#### 3.3.1. Neuron Cell

Hippocampal neurons are part of the brain’s limbic system and play a crucial role in learning, memory, and spatial orientation [90]. Nocturnal CIH is the primary pathophysiological process in OSA, shown to cause hippocampal neuronal damage and central nervous system dysfunction. Animal experiments have indicated that CIH exposure leads to increased expression of NLRP3 and pro-inflammatory cytokines in the hippocampal region of rats, while silencing NLRP3 downregulates activated Caspase1, GSDMD, and pro-inflammatory cytokine levels, thereby inhibiting hippocampal neuronal pyroptosis in CIH-exposed rats and improving their cognitive impairments [91]. However, anatomical studies of both AD and non-AD deceased patients have revealed the absence of ASC and Caspase1 in neurons of the first sector of the cornu ammonis (CA1) of the hippocampus and the temporal cortex. Instead, Caspase4 can be detected, suggesting that hippocampal neurons may undergo pyroptosis via a non-canonical pathway [92]. As for interspecies differences, future studies may need to explore the consistency of Caspase1 expression sites in the brain. Additionally, it remains to be investigated whether other cells in the hippocampus, such as immune cells, interfere with Caspase1 expression, requiring further validation of the specific cell types undergoing pyroptosis. Another characteristic of OSA, SD, can also increase levels of hippocampal neuronal pyroptosis, and this effect can be reversed upon sleep recovery [89,93]. Research indicates that the P38 and ERK-MAPK pathways, rather than the AKT pathway, mediate SD-induced activation of the NLRP3/pyroptosis axis. However, the specific activation mechanisms remain unclear [89]. Additionally, research reports that SD treatment can also activate NLRP1 and NLRC4 inflammasomes, leading to hippocampal neuronal pyroptosis and impaired spatial memory in mice [19]. Furthermore, GSDME-mediated pyroptosis has been demonstrated to occur in hippocampal neurons [94], but whether this pathway can be activated by OSA-related CIH or SD remains unclear.

In summary, there is still controversy regarding which pathways of pyroptosis neurons undergo and which inflammasomes are activated during this process. It should also be noted that some studies have not conclusively identified the cell types undergoing pyroptosis in the hippocampal region, often presuming them to be neurons without sufficient evidence. Future research should utilize more cell-specific markers or techniques such as single-cell transcriptomics to accurately identify the specific cell types involved.

#### 3.3.2. Microglia

Microglia contribute to the formation of normal neuronal connectivity, regulate central nervous system development, maintain homeostasis [95], and influence cognitive functions such as learning and memory [96]. However, with changes in the brain microenvironment, microglia can transition from a resting state to an amoeboid activated state, secreting large amounts of pro-inflammatory mediators and leading to neuronal damage [97]. Therefore, microglia play a dual role as both instigators of brain injury and guardians of brain homeostasis, crucially contributing to neuroprotection and neurodegenerative diseases [98]. Microglia in the hippocampal CA1 region and temporal cortex express all proteins involved in NLRP3 inflammasome assembly (ASC, NLRP3, and Caspase1), as well as the pyroptosis effector protein GSDMD [92]. At the same time, numerous studies have indicated that prolonged ischemia-hypoxia can induce microglia to undergo pyroptosis via the NLRP3/Caspase1/GSDMD axis [99,100,101]. However, in contrast to the above scenario, although exposure to CIH increases ROS production, activates microglial NLRP3 inflammasomes via the NF-κB signaling pathway, and promotes IL-1β release in a GSDMD-dependent manner, interestingly, it also appears to cause an increase in the number of microglia in the mouse hippocampal area. Under CIH conditions, GSDMD-NT in microglia likely serves as a protein secretion channel rather than a pyroptosis promoter [102], suggesting microglial “hyperactivation”. This hyperactivated state, with IL-1β release, may impair CA3-CA1 synaptic plasticity in the hippocampus by targeting IL-1 receptors in neurons, thereby disrupting spatial learning and memory. CIH-related activation of microglial NLRP3 inflammasomes is accompanied by enhanced mitochondrial autophagy mediated by Parkin. Damaged mitochondria release mtROS and mtDNA, further promoting assembly of the NLRP3 inflammasome complex [103].

These study findings suggest that differential cellular outcomes resulting from inflammasome activation may be associated with different hypoxia patterns. GSDMD-NT, acting as a secretion pathway for inflammatory factors, participates in CIH-induced cognitive impairment through a non-pyroptotic mechanism. Given the close association of “hyperactivation” of microglia with mitochondrial damage, future research should delve into the functional status of microglial mitochondria under various hypoxic conditions. This approach could help elucidate the outcomes of microglia in patients with OSA.

#### 3.3.3. Astrocyte

Astrocytes, the most abundant glial cells in the CNS, play vital roles in maintaining homeostasis and are closely linked to cognitive functions. For example, astrocytes can participate in clearance of Aβ both in vivo and in vitro. However, under certain inflammatory conditions, astrocytes can produce Aβ, thereby promoting the progression of AD [104]. Animal experiments have revealed that CIH induces enhanced lactate dehydrogenase (LDH) release and ROS levels, leading to astrocyte activation. Concurrently, there is upregulation of GSDMD, Caspase1, and IL-1β expression, accompanied by a decrease in viable cell count, suggesting increased pyroptosis of astrocytes. Further studies indicate that astrocytic pyroptosis is associated with the HIF1α/*miR-210* axis [88]. The brain region involved in the aforementioned studies was the cerebral cortex rather than the hippocampus. Studies have shown that astrocytes in the hippocampal CA1 region and temporal cortex do not express ASC, Caspase1, or IL-18. However, GSDMD-NT and Caspase8 were detected, and only physiological levels of Caspase3 were observed, suggesting the absence of apoptotic processes. This indicates that hippocampal astrocytes may undergo pyroptosis through a Caspase8-dependent pathway [92].

Therefore, the types of pyroptosis-related proteins expressed and the type of pyroptosis executed by astrocytes in the hippocampus versus the cerebral cortex may differ. Currently, there is limited research on pyroptosis of hippocampal astrocytes in OSA patients. Further research is required to clarify the response of hippocampal astrocytes under CIH conditions and their effects on neurons.

In summary, CIH and SD associated with OSA can induce various types of pyroptosis in hippocampal neurons. The activation and pyroptosis of immune cells in the hippocampus can release pro-inflammatory factors, exacerbating tissue inflammation and further activating neuronal pyroptosis. The diverse forms of nerve cell pyroptosis attract surrounding immune cells, complicating immune responses within the CNS [105]. Inhibiting nerve cell pyroptosis in OSA patients holds promise as a way to alleviate or delay the progression of neurocognitive diseases, although much remains unknown in this field. Future research could focus on the following aspects: validating the expression of pyroptosis-related proteins and types of pyroptosis across different types of nerve cells; investigating changes in mitochondrial function under hypoxic conditions and their impact on nerve cell pyroptosis; further exploring regulatory pathways of pyroptosis; and studying the effects of inflammasome activation on cellular interactions, particularly the interplay between immune cells and neurons.

### 3.4. The Promotive Role of Pyroptosis in Skin Diseases of OSA Patients

Psoriasis is an immune-related chronic inflammatory skin disease, clinically characterized by localized or widespread erythema, papules, and scaling [106]. Research indicates a bidirectional relationship between OSA and psoriasis [107]. Some cohort studies have shown that untreated OSA patients have a 62% to 130% increased risk of developing psoriasis compared to non-OSA patients, which may be related to the activation of inflammatory pathways [107,108,109]. Recent studies have confirmed that pyroptosis is one of the mechanisms involved in the pathogenesis of psoriasis [110]. Given that OSA can induce pyroptosis through CIH and SD, it is hypothesized that pyroptosis may be the mediation between OSA and psoriasis.

Tissue-level analysis has revealed abnormal expression of activated Caspase1 and GSDMD-NT in psoriatic lesions, compared to no expression in normal skin tissue. In the psoriasis-like immune microenvironment, simulated by pro-inflammatory factors such as TNFα, primary human epidermal keratinocytes exhibit typical pyroptotic morphology characterized by cell swelling and bubbling. Concurrently, levels of Caspase1 and GSDMD-NT in the supernatant increase, along with elevated expression of cell death markers, suggesting that GSDMD-mediated pyroptosis of epidermal keratinocytes is initiated in psoriatic lesions [111]. Analysis of differentially expressed genes in psoriatic lesions shows that NLRP3 holds significant diagnostic value for psoriasis. Immunohistochemical studies in mouse models of psoriasis have also confirmed that NLRP3 expression levels are significantly higher than those in control groups [112]. However, the upstream regulatory mechanisms governing pyroptosis in epidermal keratinocytes remain unclear. Some scholars suggest a potential association with NF-kB activation, but this has yet to be validated. Furthermore, recent studies have found that TNFα can induce keratinocyte pyroptosis through the Caspase3/GSDME pathway, providing new insights into the progression of psoriasis [113]. This further highlights the significant role of TNFα in initiating pyroptosis in epidermal keratinocytes. There is currently no direct evidence regarding how OSA induces psoriasis through pyroptosis. Given that TNFα expression is significantly upregulated in OSA patients [114], we hypothesize that OSA may initiate pyroptosis in epidermal keratinocytes through changes in TNFα levels, contributing to the pathology of psoriasis. Additionally, hypoxia may lead to mitochondrial dysfunction and increased ROS levels, which can activate the NLRP3 inflammasome and trigger pyroptosis in keratinocytes [115].

Additionally, pyroptosis plays an important role in the pathogenesis of systemic lupus erythematosus (SLE) and atopic dermatitis (AD). There is an association between OSA and the accumulation of damage and disease activity in SLE [116], as well as an increased risk of developing AD [117]. However, there is limited research on the association between OSA and these skin diseases. The causal relationship between OSA, SLE, and AD remains unclear and requires further investigation for confirmation.

**Figure 2:**
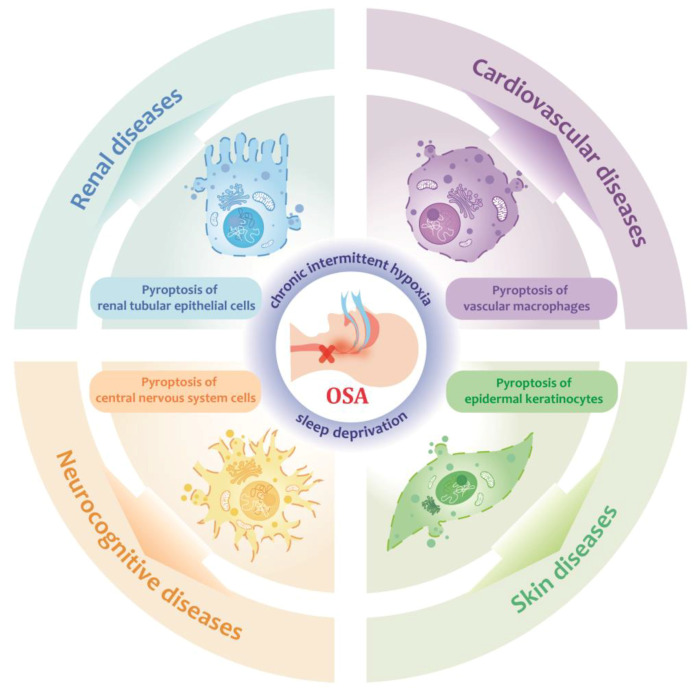
OSA promotes systemic complications through pyroptosis.

Integrating current research, OSA may promote pyroptosis in epidermal keratinocytes through TNFα overexpression and increased ROS production, which could be a key factor in the higher incidence of psoriasis among OSA patients. Although the connections between OSA and other inflammatory skin diseases require further investigation, pyroptosis provides a potential avenue for understanding the interactions more comprehensively. An overview of how OSA induces systemic complications by triggering pyroptosis in certain cells is presented in Figure 2 and Table 1.

## 4. Strategies to Inhibit Pyroptosis in OSA Patients

### 4.1. Inhibiting NLRP3 Inflammasome Activation

While other types of inflammasome activation may exist in patients with OSA, based on our current understanding from existing research, NLRP3 inflammasome activation is predominantly triggered under conditions of CIH and SD. Therefore, inhibiting NLRP3 inflammasome activation undoubtedly becomes one of the strategies to suppress OSA-related pyroptosis.

As mentioned earlier, CIH and SD elevate intracellular ROS levels, which may serve as secondary signals for inflammasome activation, either directly or by promoting oxLDL formation. Continuous positive airway pressure (CPAP) is the first-line treatment for OSA, reducing respiratory events, and improving sleep fragmentation, excessive daytime sleepiness, and daytime functioning [118]. Oxidative stress results from an imbalance between ROS production and antioxidant defenses [119]. Research has found that long-term CPAP therapy effectively reduces plasma oxidative stress markers and inflammatory factors in OSA patients [120,121,122,123] and partially improves antioxidant defense capabilities [124]. However, short-term CPAP therapy does not show these effects [123,125]. Therefore, CPAP therapy may potentially serve as one of the strategies to inhibit pyroptosis in OSA patients, but attention must be paid to the impact of patient compliance and treatment duration on therapeutic outcomes. Nuclear factor erythroid-2 related factor 2 (Nrf2) is a crucial transcription factor in antioxidant pathways, inhibiting ROS-induced NLRP3 inflammasome activation [126]. Further research indicates that Salvianolic acid B (SalB) inhibits the expression of Kelch-like ECH-associated protein 1 (Keap1), thereby activating Nrf2 nuclear translocation and inducing heme-oxygenase-1 (HO-1). This activation effectively suppresses ROS release, reduces oxidative stress levels in vivo, and consequently prevents NLRP3 activation, improving tubular epithelial cell necroptosis in AKI patients [126].

The NF-κB signaling pathway is the main mechanism driving NLRP3 inflammasome activation. Thus, inhibiting NF-κB could be an effective strategy to suppress NLRP3 inflammasome activation. Safe doses of arsenic trioxide (ATO) prevent foam cell formation by reducing CD36-mediated cholesterol uptake both in vivo and in vitro, downregulating TLR4 expression, decreasing IκBα degradation, and inhibiting p65 nuclear translocation, ultimately suppressing NLRP3 inflammasome activation and macrophage pyroptosis [127]. These findings suggest that using safe doses of ATO may represent a potential strategy for treating AS associated with OSA. Additionally, SalB [128], biochanin A (BCA) [129], Apigenin (API) [130], Salidroside (Sal) [131], and Kaperfol (KAE) [132] can also modulate the NF-κB/NLRP3 pathway, improving pyroptosis under oxidative stress. These drugs may exert their effects by reversing NF-κB nuclear translocation, inhibiting IκB phosphorylation, and other related processes.

Modafinil (MOD) is a potent wake-promoting drug that improves excessive daytime sleepiness, enhances attention, learning ability, and cognitive function [133]; it is used as adjunctive therapy in the clinical management of OSA [134]. MOD exhibits anti-inflammatory activity by reducing oxidative stress and the production of pro-inflammatory factors, and it also possesses neuroprotective effects [135,136]. MOD can alleviate hippocampal neuronal pyroptosis mediated by NLRs inflammasomes and consequent neuroinflammation under SD conditions, improving learning and memory in SD mice. However, its action on inflammasomes has not been further studied experimentally [19]. Despite these considerations, MOD represents a promising adjunct anti-inflammatory agent for cognitive disorders related to OSA. Yet, increasing evidence suggests its potential for addiction [137], necessitating cautious use with close monitoring of patient responses and potential addictive risks in clinical practice to ensure safety and efficacy during treatment.

Overall, among the control strategies targeting pyroptosis in OSA patients, inhibiting the activation of NLRP3 inflammasomes by suppressing ROS production and NF-κB signaling pathway activation appears feasible. However, besides CPAP therapy showing more definitive efficacy, whether drugs can truly be effective in human OSA requires further investigation. Moreover, inhibiting NLRP3 inflammasomes also presents certain drawbacks, such as incomplete pyroptosis inhibition and potential impacts on normal physiological functions post-NLRP3 inflammasome activation. Therefore, the development of drugs targeting precise inhibition of downstream execution proteins in pyroptosis holds broad application prospects.

### 4.2. Inhibiting GSDMD Cleavage and Pore Formation

As GSDMD is the final effector protein of pyroptosis, inhibiting GSDMD can suppress the activation of all inflammasome pathways triggered by various stimuli and reduce the release of pro-inflammatory factors during pyroptosis [138]. Currently, the primary strategies for inhibiting GSDMD focus on two aspects: inhibiting GSDMD cleavage and inhibiting GSDMD-NT membrane translocation.

Dimethyl fumarate (DMF) is a U.S. Food and Drug Administration (FDA)-approved drug for multiple sclerosis (MS), exerting anti-pyroptotic effects by limiting GSDMD cleavage. DMF reacts with the Cys191 residue of GSDMD (corresponding to Cys192 in mice) to form S-(2-succinyl)-cysteine, thereby succinylating GSDMD. This succinylation prevents its interaction with Caspase1, thereby restricting its cleavage capability. Following GSDMD knockout, the Cys45 site of GSDME can also be modified to inhibit its function [139].

Recent studies indicate that palmitoylation of Cys191 in GSDMD is essential for membrane translocation and pore formation [33,34]. This Cys residue is conserved in GSDMD but absent in other gasdermin family members [138]. Disulfiram (DSF) is a medication used to treat chronic alcohol dependence by inhibiting liver aldehyde dehydrogenase. It has also been discovered to have therapeutic potential in cancer treatment [140]. In terms of inflammation inhibition, DSF directly targets and covalently modifies Cys191, inhibiting its palmitoylation and preventing GSDMD-NT membrane translocation, thereby blocking pore formation. During this process, DSF still allows pro-inflammatory cytokine processing but prevents their release into the extracellular space [138]. Necrosulfonamide (NSA), another GSDMD inhibitor with effects similar to DSF, exerts anti-pyroptotic activity by binding to Cys191, without impacting TLR signaling, inflammasome activation, GSDMD cleavage, or cytokine maturation [141]. Additionally, NSA itself is an MLKL inhibitor, exerting beneficial effects in controlling necroptotic induced by hypoxia.

NU6300 employs a dual mechanism to inhibit pyroptosis, targeting both the cleavage and palmitoylation of GSDMD by covalently modifying its Cys191 residue. The potential palmitoylation of both full-length GSDMD and GSDMD-NT, combined with the inability of specific palmitoylation inhibitors to block Caspase1-mediated GSDMD cleavage, suggests that NU6300 inhibits GSDMD cleavage and palmitoylation via two independent mechanisms. Simultaneous action at both stages contributes to comprehensive suppression of cell pyroptosis. Additionally, NU6300 exhibits strong selectivity towards GSDMD, as it does not prevent ASC oligomerization or Caspase1 activation in AIM2 and NLRC4 inflammasomes. Similar to DSF and NSA, NU6300 demonstrates feedback inhibition of the NLRP3 inflammasome, possibly because K+ efflux was blocked due to abolishment of GSDMD pore-forming capability, thereby suppressing NLRP3 inflammasome activation [142].

The use of GSDMD inhibitors represents a particularly attractive therapeutic approach, capable of profoundly inhibiting pyroptosis at the protein level without affecting other physiological functions of the NLRP3 inflammasome. While NU6300 offers new insights into inhibiting pyroptosis in OSA patients through its multifaceted actions, animal studies indicate a narrow safety margin for cardiac toxicity and low systemic exposure, necessitating further structural optimization. In contrast, the significant advantage of using DSF and DMF lies in their FDA-approved status, ensuring established safety profiles. Currently, there is a lack of research specific to OSA patients, necessitating further animal and clinical trials to validate the efficacy of these strategies in treating pyroptosis in OSA patients. This effort aims to provide additional beneficial information and options for clinical treatment.

### 4.3. Regulating miRNA

MiRNAs are endogenous, short non-coding RNAs in eukaryotes, typically 17–25 nucleotides long. They bind to the 3′UTR of target mRNA, inhibiting target gene expression. In recent years, miRNA has increasingly garnered attention in OSA-related research. High-throughput sequencing of plasma exosomal miRNAs from severe OSA patients has revealed 28 differentially expressed miRNAs compared to non-OSA controls. These miRNAs are enriched in pathways associated with viral infectious diseases, tumors, neurodegenerative diseases, and cardiovascular disorders. Additionally, OSA patient-derived plasma exosomes can induce pyroptosis in hippocampal cells [143]. These findings suggest that miRNAs may serve as important diagnostic biomarkers and therapeutic targets for OSA and its comorbidities.

*MiR-155* is a hypoxia-related miRNA [144]. Under CIH conditions, *miR-155* can act as a positive regulator of RTEC pyroptosis by inhibiting the FoxO3a/ARC signaling pathway [65,66]. Simultaneously, *miR-155* has been shown to play a role in the pathogenesis of Alzheimer’s disease in mice. Microglial deletion of *miR-155* induces a pre-microglia neurodegenerative phenotype (MGnD) activation state, enhances microglial phagocytosis and amyloid plaque compaction, preserves synaptic integrity, and improves cognitive function in mice. However, its role and cell specificity in the human brain remain poorly understood [145]. Therefore, targeted inhibition of *miR-155* may have beneficial effects in addressing both renal damage and cognitive disorders associated with OSA. Besides the inhibitory effect of GSDMD, DMF also downregulates the expression of *miR-155* [146]. Additionally, semaglutide [147], boswellic acids (BA) [148], mulberry fruit extract (MFE) [149], and Shenlian extract [150] have been reported to downregulate levels of *miR-155*. However, these studies did not investigate pyroptosis effects related to *miR-155*.

*MiR-210* is also a hypoxia-inducible miRNA, with induction levels higher during intermittent hypoxia compared to chronic hypoxia, and it is directly regulated by HIF1α [151]. Data collected from two independent cohorts indicate that serum *miR-210* levels are higher in individuals with OSA compared to healthy controls, and significantly positively correlate with AHI, suggesting that serum *miR-210* concentration depends on the severity of OSA. Multivariate analysis reveals that both BMI and *miR-210* are common predictive factors for OSA [152]. *MiR-210* plays a crucial role in astrocyte pyroptosis associated with CIH. Overexpression of *miR-210* increases the expression of Caspase1, GSDMD, and IL-1β, indicating elevated pyroptosis levels, and promotes LDH release, as well as increasing intracellular ROS levels. Paeoniflorin can alleviate hypoxia-induced astrocytic pyroptosis by suppressing the expression of HIF1α, thereby reducing *miR-210* expression [88].

*MiR-223-3p* inhibits pyroptosis in various cells, such as endothelial cells, gastric cancer cells, synoviocytes, degenerative nucleus pulposus cells, and macrophages, by directly targeting NLRP3 [153,154,155,156]. Animal studies show that CIH reduces *miR-223-3p* expression in the rat hippocampus, while its overexpression suppresses neuronal pyroptosis, lowers inflammatory factors, and improves cognitive deficits. However, further research is needed to determine whether this target is applicable to humans. Of particular interest, research indicates that exogenous miRNA can remain stable in the digestive tract and be absorbed, thereby modulating host gene expression. SID-1 transmembrane family member 1 (SIDT1) on the surface of gastric pit cells in animal models plays a critical role in the uptake of dietary miRNAs [157]. This finding theoretically supports the development of oral miRNA formulations for the treatment of pyroptosis-related diseases.

MiRNAs can function both as positive regulators to promote pyroptosis, facilitating the progression of multi-system complications in OSA patients, and as negative regulators to downregulate pyroptosis-associated gene expression, thereby improving pyroptosis-related diseases. Future research can focus on identifying additional miRNA targets that regulate pyroptosis, facilitating the development of targeted regulatory strategies. Additionally, it is essential to further validate the feasibility of oral formulations of exogenous miRNAs for clinical applications and explore effective administration routes to provide more personalized treatment options for OSA patients.

**Figure 3:**
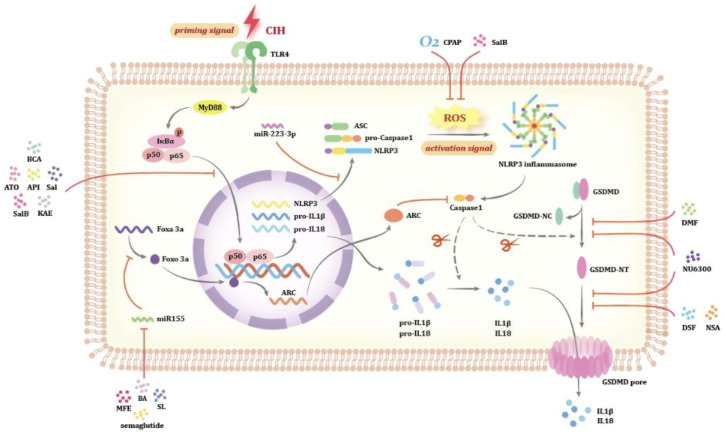
Mechanisms and targets for inhibiting pyroptosis in OSA patients. CPAP and SalB: inhibit ROS release. BCA, ATO, API, Sal, SalB, KAE: inhibit the NF-κB/NLRP3 axis activation. DMF, NU6300: inhibit the cleavage of GSDMD. DSF, NSA: prevent the membrane translocation of GSDMD-NT. MFE, BA, SL, semaglutide: downregulate the levels of *miR-155*. The red arrow represents the inhibitory effect of the molecules.

In summary, inhibiting OSA-related pyroptosis at various levels appears feasible (Figure 3), but most drugs lack clinical trials to validate their efficacy. Furthermore, determining the optimal stage of pyroptosis inhibition to achieve the best therapeutic efficacy, maximum safety, and minimal potential adverse effects requires further research to assist in selecting the optimal treatment strategy.

## 5. Conclusions and Outlook

OSA is a sleep-related breathing disorder characterized by CIH and SD, often linked to various complications. Pyroptosis, a Caspase-dependent inflammatory form of programmed cell death, contributes to tissue inflammation. Research shows that pyroptosis is crucial in renal diseases, cardiovascular diseases, neurocognitive diseases, and skin diseases. OSA mainly triggers NLRP3 inflammasome-mediated pyroptosis via the canonical pathway. CIH activates TLR4, driving NLRP3 transcription and pro-inflammatory cytokine expression through the NF-κB pathway. ROS and oxLDL generated by this stimulation act as secondary signals for NLRP3 inflammasome activation. MiRNAs also play a significant regulatory role in this pathway. Therefore, inhibiting NLRP3 inflammasome activation, suppressing GSDMD cleavage or membrane pore formation, and modulating miRNA levels could serve as effective strategies to inhibit OSA-related pyroptosis.

It is important to note that while many studies report that OSA-related factors like CIH and SD can induce pyroptosis, some lack assessments of cell proliferation activity and/or specific cellular morphological evidence. Instead, they rely solely on the activation of pyroptosis-related proteins and elevated inflammatory factor expression to confirm pyroptosis. Given GSDMD’s non-pyroptotic roles, it remains unclear whether cell death occurred or what form it took in these studies. Additionally, most studies use CIH animal models to simulate hypoxia in moderate to severe OSA. However, key differences exist between CIH models and OSA patients. For instance, OSA patients typically exhibit normal or intermittently elevated arterial CO_2_ levels, while CIH animals may show reduced arterial CO2 due to hyperventilation triggered by hypoxia. This suggests that future research should establish animal models of CIH accompanied by hypercapnia to more accurately simulate the pathophysiological conditions of OSA patients [102].

Another important point is, although pyroptosis is involved in the occurrence and development of OSA-related diseases, it also plays a crucial role in maintaining normal physiological functions. As a component of the innate immune system, pyroptosis is essential for the clearance of infections and tumor cells. Pyroptosis exposes pathogens to extracellular defenses, such as neutrophils, by lysing the cell membrane, thereby eliminating pathogens that can evade macrophage antibacterial activity and replicate within them [158]. The IL-1β and IL-18 produced during pyroptosis also play significant roles in immune defense. IL-1β rapidly recruits neutrophils to sites of inflammation, activates endothelial adhesion molecules, induces cytokines and chemokines, triggers fever responses, and stimulates specific adaptive immunity, such as Th17 responses, contributing to anti-infection mechanisms [159]. IL-18, as an immunomodulatory cytokine, induces NK cells to produce IFNγ and promotes T cells to produce IL-17 [160]. Additionally, studies have shown that the executioner protein of pyroptosis, gasdermin, plays a role in regulating development and maintaining tissue homeostasis. For example, GSDMD can generate a non-pyroptotic 20-kDa fragment (p20) through cleavage by RIPK1 and Caspase8/3, which limits the maturation and secretion of osteoclast lysosomes, thereby preventing excessive bone resorption [161]. GSDMB can also translocate in its full-length form to the intestinal epithelial cell membrane, regulating cell proliferation, migration, and adhesion to promote epithelial repair and reconstruction [162]. Therefore, we must pay attention to the adverse effects of inhibiting pyroptosis and strive to minimize them. Given that current research suggests that GSDMD-mediated pyroptosis is a major promoter of OSA-related diseases, targeting the inhibition of GSDMD may help avoid the negative effects associated with inhibiting other gasdermin proteins, while still preserving GSDMD’s normal physiological functions. Exploring the possibility of partially inhibiting pyroptosis to a certain extent, or selectively inhibiting pyroptosis in specific organs or cells, may be a direction worth considering in the future.

While this article primarily discusses renal diseases, cardiovascular diseases, neurocognitive diseases, and skin diseases associated with OSA, the spectrum of diseases accompanying OSA is by no means limited to these four. The bidirectional relationship between OSA and hypertension is well-established. Patients with OSA-related hypertension have a higher risk of target organ damage compared to those with hypertension alone [163,164]. OSA is also an independent risk factor for type 2 diabetes mellitus and contributes to diabetic peripheral neuropathy [165,166]. Additionally, OSA may elevate the risk of lung and colorectal cancers. OSA-related CIH and SD may be involved in the pathogenesis of tumors and affect prognosis [167,168]. In clinical practice, vigilance is required surrounding the occurrence of multi-organ complications in OSA patients, and further exploration into the role of cell pyroptosis in these diseases is warranted. Research on the mechanisms linking OSA to its complications may reveal new therapeutic targets and more effective treatments.

## Figures and Tables

**Table 1:** Overview of pyroptosis in certain cells of OSA patients.

Disease	Affected Organs	Pyroptotic Cells	Environment	Pyroptosis Type			Regulatory Axis
				PRRs	Caspases	GSDMs	
Acute kidney injury	Kidney	Renal tubular epithelial cell	CIH	NLRP3	1, 11, * 3	D, * E	TLR4/MyD88/NF-κB, CHOP/Caspase-11, *miR-155*/FoxO3a/ARC
Atherosclerosis	Blood vessel	Macrophage	CIH	NLRP3	1	D	HIF-1α/NF-κB, CD36/TLR4-TLR6/NF-κB
Dementia/mild cognitive impairment	Hippocampus	Neuron cell	CIH	NLRP3	1/* 4	D	*miR-223-3p*/NLRP3
			SD	NLRP1, 3, NLRC 4	1	D, * E	P38 and ERK MAPKs
		# Microglia	CIH	NLRP3	1	D	ROS/NF-κB
		Astrocyte	-	-	* 8	* D	HIF1α/*miR-210*
Psoriasis	Skin	Keratinocyte	TNFα, CIH	NLRP3	1, 3	D, E	* NF-κB

Notes: * represents proteins or signaling axes that may be involved in the process of pyroptosis in corresponding OSA-related systemic complications, but their actual effects need to be validated through in vivo and in vitro experiments. # represents that under CIH conditions, GSDMD-NT in microglia likely serves as a protein secretion channel rather than a pyroptosis promoter.
